# Small Nucleolar RNA and C/D Box 15B Regulate the TRIM25/P53 Complex to Promote the Development of Endometrial Cancer

**DOI:** 10.1155/2022/7762708

**Published:** 2022-09-26

**Authors:** Jing-tao Wen, Xi Chen, Xin Liu, Bu-min Xie, Jing-wen Chen, Hong-lei Qin, Yang Zhao

**Affiliations:** Department of Obstetrics and Gynecology, Department of Gynecologic Oncology Research Office, Guangdong Provincial Key Laboratory of Major Obstetric Diseases, The Third Affiliated Hospital of Guangzhou Medical University, Guangzhou 510150, China

## Abstract

**Background:**

Endometrial cancer is associated with a high mortality rate, which warrants the identification of novel diagnostic markers and therapeutic targets. The aim of this study is to evaluate the role of SNORD15B in the development of endometrial cancer and explore the potential underlying mechanisms.

**Methods:**

Bioinformatics was used to analyze the expression level and prognostic relevance of SNORD15B in endometrial cancer. The Ishikawa and HEC-1B cells were respectively transfected with SNORD15B expression plasmid and an antisense oligonucleotide, or the corresponding empty vector and a nonspecific sequence. The malignant phenotype of the suitably transfected cells was assessed by standard *in vitro* functional assays and the establishment of *in vivo* xenografts. The expression levels of the specific markers were analyzed with RT-qPCR and western blotting. The subcellular localization of P53 was determined by analyzing the nuclear and cytoplasmic fractions. RIP, Co-IP, and immunohistochemistry were performed as per standard protocols.

**Results:**

SNORD15B was overexpressed in the endometrial cancer tissues and correlated to a poor prognosis. Ectopic expression of SNORD15B in Ishikawa cells inhibited apoptosis, increased the proliferation, invasion, and migration *in vitro*, and enhanced their tumorigenicity *in vivo*. SNORD15B overexpression also upregulated TRIM25 and accelerated P53 accumulation in the cytoplasm of the endometrial cancer cells.

**Conclusion:**

SNORD15B functions as an oncogene in endometrial cancer by targeting the TRIM25/P53 complex and blocking the nuclear translocation of P53.

## 1. Introduction

Endometrial cancer is the sixth most commonly diagnosed cancer in women worldwide, and its incidence rate is increasing annually. Over 47,000 new cases of endometrial cancer were diagnosed in 2020, and the number of deaths exceeded 97,000. The mortality rate of endometrial cancer increased significantly in the United States between 2014 and 2018, with an average annual percentage change (APCC) of 2% [1, 2]. Therefore, it is vital to explore new diagnostic markers and therapeutic targets for endometrial cancer to improve the prognosis of patients.

Small nucleolar RNAs (snoRNAs) are a type of noncoding RNAs that typically consist of 60–300 nucleotides and are primarily localized in the nucleoli of eukaryotic cells. They regulate protein translation, mRNA splicing, and genome stabilization, and are thus involved in critical biological processes [3]. Based on some conserved structural elements, snoRNAs can be classified into the box H/ACA snoRNAs (SNORA), box C/D snoRNAs (SNORDs), and MRP RNAs [4, 5]. Human SNORDs are located within the introns and are released by debranching enzymes during the splicing process. The SNORDs form a complex with nucleolar protein 5 (NOP58), nonhistone chromosomal protein 2-like 1 (NHP2L1), nucleolar protein 5A (NOP56), and fibrillarin (FBL), which prevents exonuclease-mediated degradation [6–8]. While SNORDs have long been known to mediate 2′-O-ribose methylation of rRNAs, recent studies have revealed a more diverse functional repertoire, which may be associated with the occurrence and progression of cancer. For example, the deletions in the SNORD50 sequence are associated with breast cancer and prostate cancer [9, 10], whereas SNORD17 promotes human hepatocellular carcinoma progression [11]. Furthermore, the overexpression of SNORD1C in malignant colorectal tumors is related to a poor prognosis [12], and SNORD12B functions as an oncogene in esophageal squamous cell carcinoma [13]. According to gene expression profiling interactive analysis (GEPIA), SNORD15B is highly expressed in acute myeloid leukemia, ovarian serous cystadenocarcinoma, endometrial cancer, and uterine carcinosarcoma. Therefore, it is reasonable to surmise that SNORD15B may promote endometrial cancer development.

In this study, we confirmed the overexpression of SNORD15B in endometrial tumor tissues and found that the upregulation of SNORD15B was associated with a poor prognosis. In addition, the ectopic expression of SNORD15B in it also promoted the malignant phenotype of the endometrial cancer cells *in vivo* and *in vitro*. SNORD15B exerted its effects by preventing the nuclear translocation of the tumor suppressor P53 via the upregulation of TRIM25.

## 2. Materials and Methods

### 2.1. Bioinformatics Analysis

The expression data of SNORD15B in endometrial cancer and normal endometrial samples were retrieved from GEPIA (https://gepia2.cancer-pku.cn, version 2). The correlation between SNORD15B expression and patient prognosis was analyzed using the Kaplan-Meier plotter (https://kmplot.com/analysis/). The National Center of Biotechnology Information (https://blast.ncbi.nlm.nih.gov/Blast.cgi) was used to predict the base pairing of two genes.

### 2.2. Cell Culture

The endometrial cancer cell lines HEC-1B were obtained from Jennio Biotech (Guangzhou, China) and cultured in high-glucose Dulbecco's modified eagle medium (Gibco, Waltham, MA, USA). The HEC-1A and Ishikawa cell lines were purchased from the China Center for Type Culture Collection (Wuhan, China) and cultured in RPMI-1640 medium (Gibco). The cell culture media were supplemented with 10% fetal bovine serum (FBS; Gibco), 1% streptomycin, and 1% penicillin. All cell lines were cultured at 37°C under 5% CO_2_. The medium was changed every one or two days, and the adherent cells were passaged with trypsin once confluent.

### 2.3. Transfection Experiments

Antisense oligonucleotide (ASO) targeting SNORD15B and a control ASO were purchased from RiboBio Co., Ltd. (Guangzhou, China). The HEC-1B cells were transfected with the respective constructs (final concentration 25 nM) using Lipofectamine™ 3000 according to the manufacturer's protocol. Plasmids expressing SNORD15B and a vector were obtained from SyngenTech (Beijing, China). Ishikawa cells were transfected with the respective plasmids at 70% confluence using Lipofectamine™ 3000 and P3000 as per the manufacturer's instructions. The sequences of the different constructs are listed in Table 1 and Table 2.

### 2.4. Cell Viability Assay

The cells were seeded in 96-well plates at a density of 2 × 10^3^/100 *μ*L/well and allowed to adhere. Following transfection with ASO-SNORD15B or ASO-NC (empty vector), the cells were cultured for 0, 24, 48, and 72 h. At the stipulated time points, a 10 *μ*L cell counting kit reagent (Yeasen Biotech, Shanghai, China) was added to each well, and the cells were incubated for 2 h. The absorbance at 450 nm was measured using a microplate reader.

### 2.5. RT-qPCR

The total RNA was extracted from the suitably transfected cells using RNAiso plus (Takara, Japan) and reverse-transcribed using Hifair® III 1st Strand cDNA Synthesis SuperMix for qPCR (gDNA digester plus, Yeasen Biotech, Shanghai, China). RT-PCR was performed using Hieff® qPCR SYBR® green master mix (Yeasen Biotech, Shanghai, China), and the expression level of the target gene was normalized to that of U6 mRNA. The primer sequences are listed in Table 3.

### 2.6. Apoptosis Assay

HEC-1B and Ishikawa cells were seeded in 6-well plates in the 2 × 10^5^/well, transfected with the respective target ASO or plasmids, and harvested 48 h later. The adherent cells were washed twice with pre-cooled PBS, digested with EDTA-free trypsin, and then centrifuged at 1500 rpm for 5 min. After discarding the supernatant, the cells were washed twice with PBS and stained with PI and annexin-V/FITC for 15 min in the dark. The stained cells were analyzed by flow cytometry.

### 2.7. Wound Healing Assay

The cells were seeded in a 6-well plate at a density of 1 × 10^6^ cells/2 ml/well and cultured till confluent. The adherent monolayer was scratched with a sterile 200 *μ*L pipette tip and washed thrice with PBS to remove the dislodged cells. The cells were then transfected with the respective target ASO or plasmids in a low-serum (<5%) medium, and the scratched area was photographed at 0 h and 48 h. The scratched areas at each time point were measured, and the wound healing rate was quantified using the following equation: ((scratched area at 0 h − scratched area at 48 h)/scratched area at 0 h )× 100%.

### 2.8. Cell Invasion Assay

Matrigel (Becton Dickinson Labware, Bedford, MA, USA) was diluted 1 : 15 in serum-free medium to a final concentration of 8 mg/ml, and 40 *μ*l was spread evenly on the membrane lining the upper chamber of Transwell inserts (BD Bioscience, San Jose, CA, USA). The plates were incubated at 37°C for 4 h to allow the Matrigel to set, and the lower chambers of the inserts were filled with a 600 *µ*l complete medium (10% FBS-supplemented). The suitably transfected cells were seeded on the Matrigel-coated membrane in a serum-free medium at a density of 8 × 10^4^ cells/well. After 48 hours of incubation, the Transwell inserts were removed, gently washed with PBS twice, fixed with 4% paraformaldehyde for 30 minutes, washed twice with PBS again, and stained with 0.1% crystal violet. The Matrigel layer was then wiped off to remove the noninvasive cells. The membranes were cut out from the inserts, mounted on a glass slide, and observed under a microscope to count the number of invasive cells.

### 2.9. Western Blotting

The transfected cells were cultured for 48 h in 10 cm dishes, washed twice with precooled PBS, scraped, and centrifuged at 3000 rpm for 5 min. The supernatant was aspirated, and the pellets were lysed overnight in RIPA buffer (BB-3201,BestBio, Shanghai, China) supplemented with PMSF at 4°C or ultrasonicated to extract the total cellular protein. In addition, proteins were extracted from the cytoplasmic and nuclear fractions using the nuclear and cytoplasmic protein extraction kit (P0028, Beyotime biotechnology, China). The protein content in the various lysates was measured using the bicinchoninic acid (BCA) reagent (P0011, Beyotime biotechnology, China). Equal amounts of proteins per sample were diluted with PBS and 5× loading buffer to the final concentration of 3 *µ*g/*µ*l and denatured for 5 min at 95°C. The proteins were separated by SDS-PAGE and transferred onto a PVDF membrane. After blocking with 3% bovine serum albumin (BSA) at room temperature for 1.5 h, the membrane was incubated overnight with the primary antibody at 4°C. The membrane was washed the following day and incubated with the secondary antibody at room temperature for 1 h. Protein bands were analyzed using an enhanced chemiluminescence reagent. The assay was repeated three times.

### 2.10. Establishment of *In vivo* Xenografts

Female BALB/c nude mice (5 weeks, 12–15 g) were purchased from the Guangdong Medical Laboratory Animal Center and raised in an SPF environment. Each mouse was injected subcutaneously with the control or 1 × 10^7^SNORD15B-expressing Ishikawa cells (*n* = 6 each). Tumor dimensions were measured every two or three days, and the tumor volume was calculated as 1/2 × (length × width^2^). Five weeks later, the xenografts were removed, measured, and photographed. The animal experiment protocols were in accordance with the National Institutes of Health Guide for the Care and Use of Laboratory Animals and approved by the Ethics Committee of the Guangdong Medical Laboratory Animal Center (No. B202108-1).

### 2.11. RNA Immunoprecipitation (RIP)

Ishikawa cells transfected with SNORD15B or control plasmids were washed twice with precooled PBS, scraped, and centrifuged at 3000 rpm for 5 min. Following overnight lysis with PMSF-supplemented Celllysis buffer for Western and IP (P0013, Beyotime biotechnology, China) at 4°C, the cell lysates were centrifuged at 12,000 rpm for 20 min at 4°C. The supernatants were aspirated and incubated overnight with 4 *µ*g IgG (Proteintech, B900610) or 4 *µ*g of an antifibrillarin antibody (Proteintech, 16021-1-AP) on a vertical shaker at 4°C. In addition, a 20 µl aliquot was used as the input control. The following day, each sample was mixed with 50 *µ*l magnetic bead suspension and incubated at 4°C for 4 h with constant shaking. The tubes were placed against a magnetic stand, the supernatant was carefully aspirated, and the magnetic beads were washed thrice with PBS. RNA bound to the beads was extracted using 1 ml of the RNAiso plus reagent, reverse transcribed, and amplified by RT-qPCR as previously described. The primer sequences for TRIM25 are listed in Table 3.

### 2.12. Protein-Protein Immunoprecipitation (Co-IP)

The total protein was extracted from the suitably transfected Ishikawa cells as described previously and incubated with magnetic bead suspension (10 : 1) for 10 min on a shaker to bind the nonspecific proteins. Following magnetic separation, the protein content in the supernatants was measured by the BCA method, and the concentration was adjusted to 2 *µ*g/*µ*l. Each sample was split into two aliquots of 1 mg protein each (along with 20 *µ*l as the input group) and incubated overnight with 4 *µ*g IgG and 4 *µ*g anti-P53 antibody (Proteintech, 10442-1-AP), respectively, at 4°C with constant shaking. Following magnetic separation described in the previous section, each sample was denatured in 60 *µ*l 2× loading buffer for western blotting. The membrane was probed with an anti-TRIM25 antibody (Proteintech, 12573-1-AP).

### 2.13. Immunohistochemistry

The tumor xenografts were fixed in 4% paraformaldehyde, embedded in paraffin, and cut into ultrathin sections. The sections were deparaffinized with xylene and hydrated with alcohol. After antigen retrieval, the sections were quenched with 3% hydrogen peroxide for 20 min to block endogenous peroxidase activity and then incubated with 5% bovine serum albumin for 5 min to block nonspecific binding. Subsequently, the sections were incubated with an anti-TRIM25 antibody overnight at 4°C, then washed thrice with TBST (tris-buffered saline and Tween20), and probed with an HRP-conjugated antirabbit antibody for1 h. After washing thrice with TBST, the sections were developed using the chromogen 3, 3′-diaminobenzidine (DAB) and counterstained with hematoxylin. The stained sections were dehydrated through an ethanol gradient and mounted for observation.

### 2.14. Statistical Analysis

GraphPad Prism (v8.0.2) was used for statistical analysis. The data were expressed as mean ± standard error of mean (SEM) and analyzed by the *t*-test or two-way analysis of variance. *P* < 0.05 was considered statistically significant.

## 3. Results

### 3.1. SNORD15B Was Upregulated in Endometrial Cancer and Associated with Poor Outcomes

**Figure 1:**
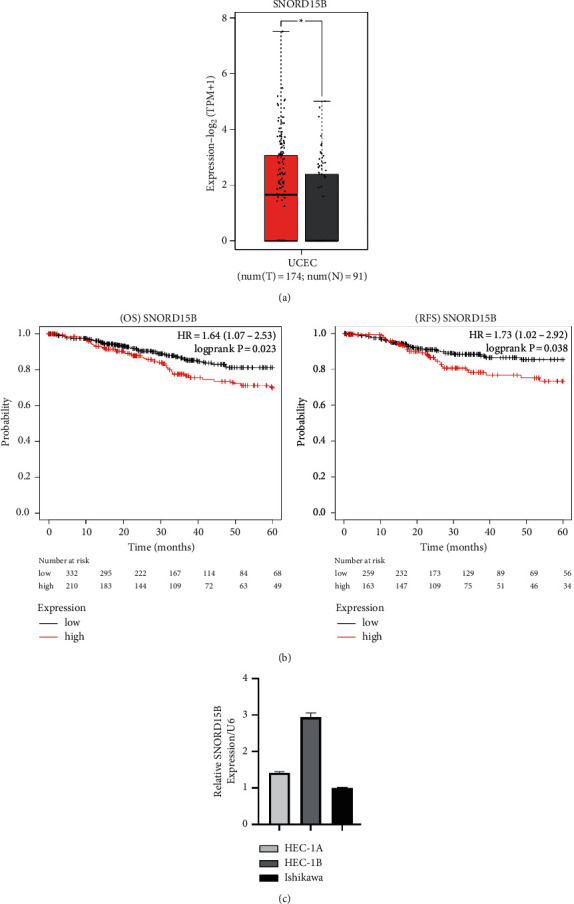
Expression level and prognostic value of SNORD15B in human endometrial cancer. (a) Expression of SNORD15B in human uterine corpus endometrial carcinoma and the normal endometrium in GEPIA2. (b) The expression level and prognostic value of SNORD15B in uterine corpus endometrial carcinoma. (c) As measured by real-time RT-PCR, the relative expression level of SNORD15B in the HEC-1A, HEC-1B, and Ishikawa cell lines.^*∗*^*P* < 0.05.

Analysis of the GEPIA2 indicated that SROND15B was significantly upregulated in the endometrial cancer tissues relative to the normal endometrial tissues (Figure 1(a)). We also analyzed the expression levels of SNORD15B in three endometrial cancer cell lines (HEC-1B, HEC-1A, and Ishikawa). As shown in Figure 1(c), the highest expression of SNORD15B was detected in the HEC-1B cells, followed by HEC-1A and Ishikawa cell lines. Kaplan-Meier analysis indicated that SNORD15B expression was negatively correlated with the 5-year over survival (OS) and 5-year recurrence-free survival (RFS) rates of endometrial cancer patients (Figure 1(b)).

### 3.2. SNORD15B Enhanced the Malignant Phenotype of Endometrial Cancer Cells *In vitro* and *In vivo*

**Figure 2:**
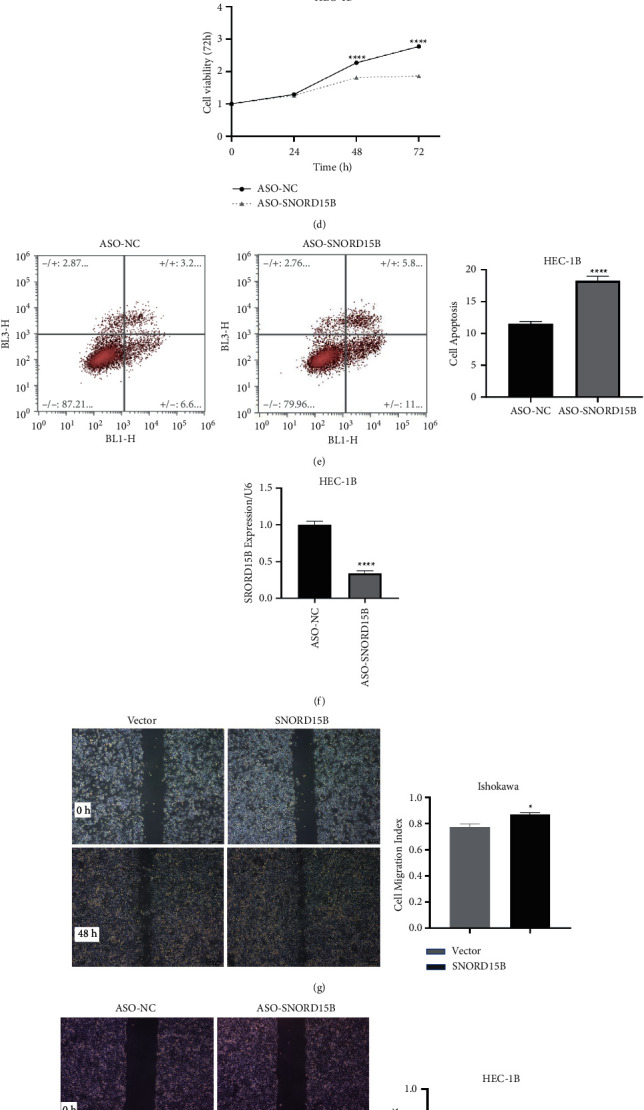
SNORD15B enhanced the malignant phenotype of endometrial cancer cells in vitro. (a, b) Overexpression of SNORD15B in Ishikawa cells (a) increased the proliferation rate and (b) inhibited apoptosis. (c) Establishment of the SNORD15B-overexpressing Ishikawa cell line. (d, e) SNORD15B knockdown in HEC-1B cells (d) decreased the proliferation rate and (e) promoted apoptosis. (f) Establishment of the SNORD15B-knockdown HEC-1B cell line. (g) Overexpression of SNORD15B increased migration of Ishikawa cells in the wound healing assay. (h) Knocking down SNORD15B inhibited the migration of HEC-1B cells. (i) Overexpression of SNORD15B increased the invasiveness of the Ishikawa cell line in the Transwell assay. (j) Knocking down SNORD15B inhibited the invasive capacity of HEC-1B cells. ^*∗*^*P* < 0.05, ^*∗∗*^*P* < 0.01, ^*∗∗∗*^*P* < 0.001, and ^*∗∗∗∗*^*P* < 0.0001.

Based on the expression level of SNORD15B in endometrial cancer cell lines, we knocked down SNORD15B in the HEC-1B cells and overexpressed it in the Ishikawa cells. The overexpression of SNORD15B enhanced the proliferation of Ishikawa cells (Figure 2(a)) and significantly reduced the apoptosis rates (Figure 2(b)). In contrast, SNORD15B knockdown had an inhibitory effect on the proliferation rates of HEC-1B cells (Figure 2(d)) and increased the proportion of apoptotic cells (Figure 2(e)). We also evaluated the impact of SNORD15B on the migration ability of the cells and found that overexpression of SNORD15B enhanced the migration and invasion rates *in vitro* (Figures 2(g) and 2(i)), whereas its knockdown had the opposite effect (Figures 2(h) and 2(j)).

**Figure 3:**
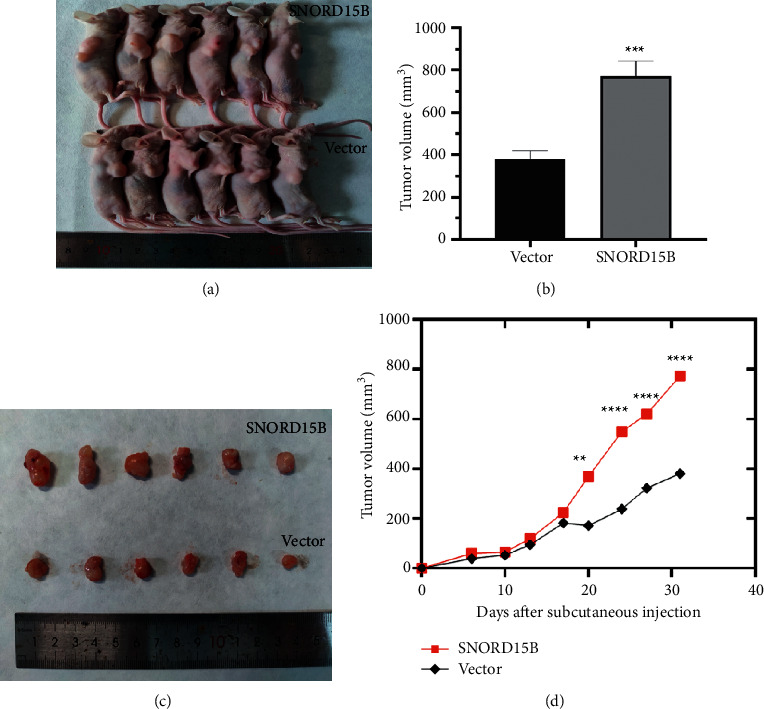
SNORD15B promoted tumor growth *in vivo*. (a–c) SNORD15B-overexpressing Ishikawa cells formed larger tumors in the nude mice compared to the control cells. (d) The growth rate of the SNORD15B-overexpressing xenografts was significantly higher compared to that of the control tumors from the third-week postinoculation. ^*∗∗*^*P* < 0.01, ^*∗∗∗*^*P* < 0.001, and ^*∗∗∗∗*^*P* < 0.0001.

To validate the *in vitro* findings, we established *in vivo* xenografts of endometrial cancer by inoculating mice with control or SNORD15B-overexpressing Ishikawa cells. As shown in Figures 3(a)–3(c), the Ishikawa cells overexpressing SNORD15B produced significantly larger tumors compared to cells transfected with the empty vector. In addition, the tumor growth rate of the SNORD15B group was significantly higher than that of the control group from the third-week postinoculation (Figure 3(d)). These findings suggested that SNORD15B functions as an oncogene in endometrial cancer and can enhance the malignant potential of the tumor cells.

### 3.3. SNORD15B Exerted Its Oncogenic Effects by Regulating the TRIM25/P53 Complex

**Figure 4:**
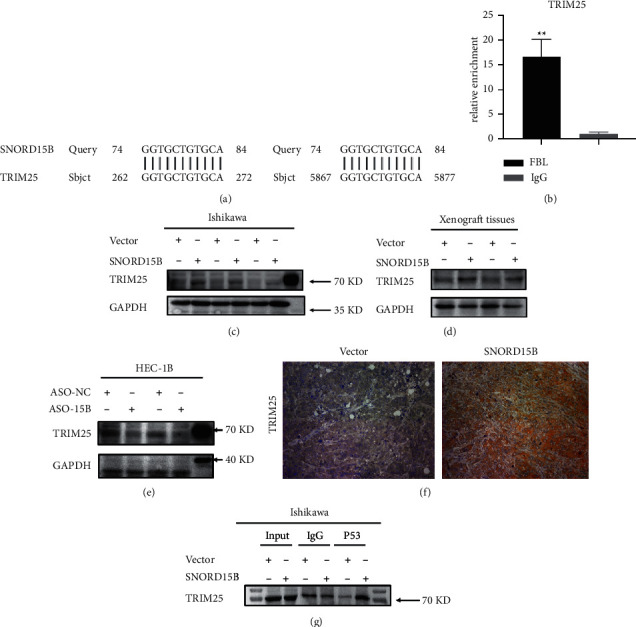
SNORD15B targets the TRIM25/P53 complex *in vitro*. (a) The predicted region of TRIM25 showed that TRIM25 was a target SNORD15B. (b) RIP experiments verified that SNORD15B interacts with TRIM25 through FBL. (c) Overexpression of SNORD15 in Ishikawa cells upregulated TRIM25. (d) Overexpression of SNORD15 upregulated TRIM25 in tumor xenografts. (e) Knockdown of SNORD15 B in HEC-1B cells downregulated TRIM25. (f) IHC staining of SNORD15B-overexpressing tumor tissues showed upregulation of TRIM25 compared to the vector control xenografts. (g) Overexpression of SNORD15B in Ishikawa cells enhanced the interaction of TRIM25 with P53. ^*∗∗*^*P* < 0.01.

**Figure 5:**
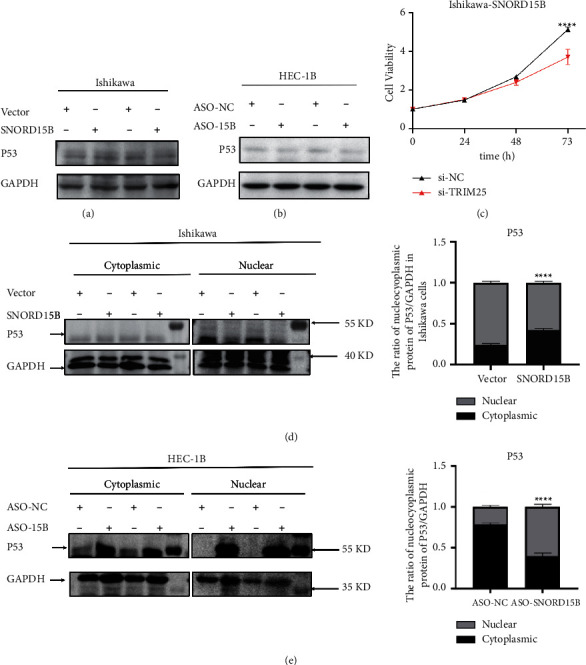
SNOND15B regulates cellular sublocalization of p53. (a-b) SNORD15B expression had no effect on the total P53 level in endometrial cancer cells. (c) Knocking down TRIM25 reversed the proliferation effect of SNORD15B. (d) Overexpression of SNORD15B in Ishikawa cells reduced P53 expression in the nucleus. (e) Knockdown of SNORD15B in HEC-1B increased P53 expression in the nucleus ^*∗∗∗∗*^*P* < 0.0001.

Small nucleolar RNAs may interact with downstream target genes in a complementary base-pairing manner. When we searched for the downstream target gene of SNORD15B, we performed a blast search. Furthermore, we found that ATP8A2, ALMS1, CACNA1s, and CUBN were the top 4 genes with complementary sequences to SNORD15B. In addition, some studies performed UV cross-linking and immunoprecipitation in Hela cells expressing T7-labeled TRIM25 and performed high-throughput sequencing on TRIM25-related RNAs. It was found that SNORD15B was one of the RNAs that interact with TRIM25 [14], but no research has been conducted on its effects. Since TRIM25 could promote the survival of cancer cells by inactivating P53 [15], we hypothesized that SNORD15B likely targeted the TRIM25/P53 complex in endometrial cancer cells. To this end, we conducted a BLAST search of the SNORD15B and TRIM25 mRNA sequences and detected 11 perfectly matched base pairs between the two (Figure 4(a)). We performed RIP experiments using FBL in Ishikawa cells overexpressing SNORD15B and found that among the five genes that have complementary base pairing with SNORD15B, only TRIM25 was linked to FBL, and the amount of TRIM25 mRNA pulled down by FBL from the SNORD15B-overexpressing Ishikawa cells was up to 16 times higher than that by IgG (Figure 4(b)). Overexpression of SNORD15B also upregulated TRIM25 protein in the endometrial cancer cells *in vitro* (Figure 4(c)) and in the tumor xenograft (Figures 4(d) and 4(f)). Conversely, knocking down SNORD15B in HEC-1B cells significantly decreased the level of TRIM25 protein (Figure 4(e)). To determine whether TRIM25 interacted with P53 in endometrial cancer cells, we performed a Co-IP assay using anti-P53 and anti-TRIM25 antibodies. As shown in Figure 4(g), a stronger coprecipitation of TRIM25 and P53 was observed in the SNORD15B-overexpressing cells than in the empty vector group, thereby confirming the interaction between TRIM25 and P53 in the presence of SNORD15B. However, the level of P53 protein was similar in both the SNORD15B-overexpressing and knockdown cells (Figures 5(a) and 5(b)), indicating that SNORD15B did not affect P53 expression. We also analyzed the nuclear and cytoplasmic fractions and detected a significantly higher expression of P53 in the cytoplasm of cells overexpressing SNORD15B compared to the empty vector control (Figure 5(d)). On the contrary, the nuclear fraction of the SNORD15B-knockdown HEC-1B cells expressed markedly higher levels of P53 compared to that of the empty vector control cells (Figure 5(e)). These findings suggested that SNORD15B might block the nuclear translocation of P53 in endometrial cancer cells by upregulating TRIM25.To further verify the role of TRIM25, we knocked down TRIM25 in Ishikawa cells that were stably transfected with SNORD15B. As shown in Figure 5(c), knocking down TRIM25 reversed the proliferation effect of SNORD15B (Figure 5(c)).

## 4. Discussion

snoRNAs are the most abundant group of noncoding RNAs involved in ribosomal biogenesis [16]. Although there is evidence that snoRNAs modulate cancer occurrence and development by targeting downstream genes [17, 18], little is known regarding the role of snoRNAs in endometrial cancer. In this study, we detected significantly higher levels of SNORD15B in endometrial cancer tissues compared to the normal endometrial tissues, and the overexpression of SNORD15B correlated with a worse prognosis. In addition, the ectopic expression of SNORD15B in the endometrial cancer cells significantly enhanced their malignant potential both *in vitro* and *in vivo*, whereas knocking down SNORD15B had opposite effects on the endometrial cancer cells *in vitro*. These findings suggest that SNORD15B is a potential oncogene in endometrial cancer.

We identified the TRIM25/P53 complex as the possible target of SNROD15 B in endometrial cancer cells. TRIM25 is a member of the triple motif (TRIM) RNA-binding protein family, and consists of an N-terminal region with the ring finger domain, one or two B-boxes (zinc-binding motif), a coiled-coil domain, and the RPY and SRPY domains (substrate-binding region) [19]. The ring finger domain has ubiquitin ligase activity, and the coiled-coil region binds to RNA and regulates gene expression post-transcriptionally [20]. TRIM25 is overexpressed in multiple malignancies, including breast cancer [19, 21, 22], lung cancer [23, 24], liver cancer [25, 26], colorectal cancer [27], prostate cancer [28], and ovarian cancer [29]. We detected complementary pairing sequences between SNORD15B and TRIM25, and further verified their direct interaction through rRNA 2′-O-methyltransferase FBL in cells overexpressing SNORD15B. Notably, the TRIM25 protein levels were significantly higher in the SNORD15B-overexpressing cells and decreased following SROND15B knockdown. A previous study showed that the ubiquitination ligase activity of TRIM25 was enhanced upon binding to SNORD15B [14]. Taken together, we can surmise that SNORD15B upregulates both TRIM25 expression and activity in endometrial cancer cells.

TRIM25 forms a complex with P53 and MDM2 in various cancer cells, such as human lung cancer cells, and the MCF7, HCT116, and H1299 cell lines, which reduces P53 expression and promotes cellular proliferation and metastasis [24]. There is evidence that TRIM25 stabilizes the P53 protein while inhibiting its activity via reduced polyubiquitination. siRNA-mediated knockdown of TRIM25 in HCT116 cells significantly increased the apoptosis rates, although this effect was obliterated in the p53-/-HCT116 cells, indicating that TRIM25 regulates apoptosis in a P53-dependent manner [15]. TRIM25 also interacts with the G3BP2 complex in prostate cancer cells and blocks the nuclear translocation of P53, resulting in decreased activity of the latter [28]. In this study, Co-IP experiment showed a direct interaction between TRIM25 and P53 in endometrial cancer cells, which was further strengthened in cells overexpressing SNORD15B. The function of P53 is regulated at multiple levels, including transcription, translation, protein stability, post-translational modifications, and cellular localization [30]. Following cytoplasmic synthesis, P53 is translocated to the nucleus, wherein it regulates the transcription of downstream target genes. We found that overexpression of SNORD15B increased cytoplasmic accumulation of P53, whereas SNORD15B knockdown increased P53 levels in the nuclear fraction. These findings suggest that SNORD15B may block the nuclear translocation of P53 in endometrial cancer cells by upregulating TRIM25, thereby inhibiting the regulatory effect of P53 on downstream genes. Our study has shown for the first time that SNORD15B possibly functions as an oncogene in endometrial cancer by regulating the TRIM25/P53 complex. Nevertheless, the mechanisms underlying the SNORD15B-mediated upregulation of TRIM25 remain unclear, which necessitates further research.

## 5. Conclusion

SNROD15B functions as an oncogene in endometrial cancer by enhancing the proliferation and migration ability of the endometrial cancer cells and inhibiting apoptosis. Mechanistically, it blocked the nuclear translocation of P53 by activating TRIM25. Thus, SNORD15B is a promising therapeutic target in endometrial and other cancers, and should be investigated further.

## Figures and Tables

**Table 1:** Plasmid information.

Carrier number	pHS-AVC-0494
Carrier information	pZdonor-CMV- SNORD15B (human NR_000025.1)-SV40 promoter-neo
Gene sequence	SNORD15B (human NR_000025.1) CTTCAGTGATGACACGATGACGAGTCAGAAAGGTCACGTCCTGCTCTTGGTCCTTGTCAGTGCCATGTTCTGTGGTGCTGTGCACGAGTTCCTTTGGCAGAAGTGTCCTATTTATTGATCGATTTAGAGGCATTTGTCTGAGAAGG

**Table 2:** Sequence report.

Product number	Product name	Target sequence
lnc6201202025322	ASO-h-SNORD15B_001	GATCGATTTAGAGGCATTTG
stB0008362A	genOFFTM st-h-TRIM25_001	GGGTCAACAGCAAGTTTGA

**Table 3:** Primer sequence.

SNORD15B	F: 5′ AAGGTCACGTCCTGCTC 3′
	R: 5′ ACAAATGCCTCTAAATCG 3′
U6	F: 5′ CTCGCTTCGGCAGCACA 3′
	R: 5′ AACGCTTCACGAATTTGCGT 3′
TRIM25	F: 5′ AGCAGCTACAACAAGAATACACG 3′
	R: 5′ GGCTCTGTTCAATCTCCTCCT 3′

## Data Availability

All data used to support the findings of this study are available from the corresponding author upon request.
